# Development of Phytocosmeceutical Microemulgel Containing Flaxseed Extract and Its In Vitro and In Vivo Characterization

**DOI:** 10.3390/pharmaceutics14081656

**Published:** 2022-08-09

**Authors:** Rabia Tasneem, Haji Muhammad Shoaib Khan, Fatima Rasool, Kashif-ur-Rehman Khan, Muhammad Umair, Tuba Esatbeyoglu, Sameh A. Korma

**Affiliations:** 1 Department of Pharmaceutics, Faculty of Pharmacy, The Islamia University of Bahawalpur, Bahawalpur 63100, Pakistan; 2 University College of Pharmacy, University of the Punjab, Lahore 54000, Pakistan; 3 Department of Pharmaceutical Chemistry, Faculty of Pharmacy, The Islamia University of Bahawalpur, Bahawalpur 63100, Pakistan; 4 Department of Food Science and Engineering, College of Chemistry and Engineering, Shenzhen University, Shenzhen 518060, China; 5 Department of Food Development and Food Quality, Institute of Food Science and Human Nutrition, Gottfried Wilhelm Leibniz University Hannover, Am Kleinen Felde 30, 30167 Hannover, Germany; 6 Department of Food Science, Faculty of Agriculture, Zagazig University, Zagazig 44519, Egypt

**Keywords:** antioxidant, cosmetic, formulation development, HPLC-UV, in vivo study, *Linum usitatissimum*, pharmaceutic, topical, phytocosmetics, radical scavenging activity

## Abstract

Antioxidants from natural sources are extensively attaining consideration to avert the skin from damage and aging caused by free radicals. Flaxseed (*Linum usitatissimum* L.), a natural therapeutic agent, was meant to be explored cosmeceutical by quantifying its potential phytoconstituents and to be incorporated into a microemulgel for topical use. Hydroalcoholic fractions (both methanolic and ethanolic; 80%) flaxseed extracts were subjected to phytochemical screening by quantifying total phenolic content (TPC), total flavonoid content (TFC), and high-performance liquid chromatography-ultraviolet (HPLC-UV), and for biological activities through 2,2-diphenyl-1-picrylhydrazyl (DPPH) assay, tyrosinase inhibition assay, and sun protection factor (SPF). Ethanolic fraction was selected for further study by TPC (18.75 mg gallic acid equivalent/g) and TFC (1.34 mg quercetin equivalent/g). HPLC-UV analysis showed the existence of benzoic, quercetin, caffeic, vanillic, *p*-coumaric, gallic, cinnamic, syringic, and sinapic acids. Biological activities showed 87.00%, 72.00%, and 21.75 values for DPPH assay, tyrosinase inhibition, and SPF assays, respectively. An oil-in-water (OW) microemulsion containing the flaxseed extract, with 99.20 nm Zeta size, −19.3 Zeta potential and 0.434 polydispersity index was developed and incorporated in Carbopol-940 gel matrix to formulate an active microemulgel with 59.15% release in in vitro studies. The successfully formulated stable active microemulgel produced statistically significant effects (*p* < 0.05), in comparison to a placebo, on skin erythema, melanin, sebum, moisture, and elasticity, in a noninvasive in vivo study performed on 13 healthy human female volunteers. Other cosmeceutical products can also be formulated from flaxseed, making it a considerable candidate for further utilization in the pharmaceutical industry.

## 1. Introduction

Among the natural and synthetic antioxidants, the former is gaining more attention, as they are considered safer due to their natural origin [1]. Plants are the major source of natural antioxidants, and their extracts are widely used in suitable solvents in the cosmetics industry [2]. Flaxseed (*Linum usitatissimum*) belongs to the family of Linaceae [3]. Flaxseeds are a rich source of natural antioxidants, as they contain significant amounts of phenolics and flavonoids along with omega-3 fatty acids, vitamins, proteins, carbohydrates, minerals, dietary fibers, and lignans [4,5]. Flaxseed is also used in many manufacturing industries including paints, printing ink, oil cloths, soaps, and varnishes [5]. Upon adding to the diet, biologically active components (polyphenols, lignans, some proteins and α linolenic acid) of flaxseed provide many health benefits, including a reduction in serum cholesterol level, slowing down the occurrence of tumor growth, and decreasing the risk of prostate, breast, and colon cancers. Lignan, an important component of flaxseed, can act as a good substitute for synthetic antioxidants [6].

When skin is revealed to ultraviolet rays, it causes the formation of reactive oxygen species, including free radicals, leading to chain reactions being a major reason for oxidative stress to the skin. The continuous increase in free radicals initiates skin damage by wrinkling, elastosis, drying, pigmentation, and photoaging [2,7]. This oxidation process is terminated or limited by antioxidants that scavenge free radicals and provide protection against aging [7]. Low molecular weight antioxidants can also be helpful in this regard. These antioxidants can be administered orally and/or can directly be applied on the skin. Nevertheless, oral administration of antioxidants may affect its effectiveness as this method limits its delivery to the skin due to physiological procedures [8]. Cosmetics containing antioxidants are a good candidate to prevent skin damage and aging by free radicals [2].

Currently, as the demand for natural antioxidants over synthetic antioxidants is rising, the cosmetics industry also prefers natural antioxidants in its products [1,2]. Phyto-cosmetics are cosmetic products of natural origin and/or containing natural ingredients (as a whole, some specific parts or extract, etc.) from plants or algae. Moreover, phyto-cosmetics can also contain extracts, essential or fixed oils, or unorganized substances such as resins and waxes that may be the active ingredients in the product [9]. The absorption (the transportation of the unmetabolized drug from the site of administration) of several cosmetic ingredients, such as moisturizers and whitening agents, through the percutaneous route is less because of the stratum corneum of skin that acts as a barrier [10]. Permeation may also be affected by other characteristics of drug molecules including their mass, size, and polarity [11]. Skin permeation of cosmetic substances through the stratum corneum is increased by different formulation approaches. According to previous research, the microemulsion is a useful formulation, as it can deliver the drug and cosmetic substances through the stratum corneum more efficiently than other conventional dosage forms because of nanosized droplets of dispersed phase that can easily cross the stratum corneum barrier [10]. Microemulsions have surfactants and cosurfactants that are good absorption enhancers, decreasing the size of the droplets as well as increasing the interfacial area [11]. Three types of microemulsion are most likely to be formed depending on the composition: oil-in-water microemulsions wherein oil droplets are dispersed in the continuous aqueous phase; water-in-oil microemulsions wherein water droplets are dispersed in the continuous oil phase; and bicontinuous microemulsions wherein microdomains of oil and water are interdispersed within the system [12,13,14].

Microemulgel, being a combination of microemulsion and gel, is a unique and novel dosage form that can cope with the problems associated with conventional dosage forms and have the advantages of both gel and microemulsion. Microemulgels have proved to improve penetration as compared to creams [15]. Thus, the goal of this investigation was to ascertain total phenolic and flavonoid contents, antioxidant activity, antityrosinase activity, sun protection factor (SPF), and quantification of polyphenols via high-performance liquid chromatography-ultraviolet (HPLC-UV) of flaxseed extract followed by the development of microemulgel incorporating the ethanol extract of flaxseeds for skincare and its in vitro evaluation by assessing pH, conductivity, and rheology at different temperatures for three months and comparison of release studies of microemulsion and microemulgel as well as its in vivo evaluation by measuring biophysical parameters of skin including erythema, melanin, sebum, moisture, and elasticity that has not been done before.

## 2. Materials and Methods

### 2.1. Material

Flaxseed was purchased from the local market of Bahawalpur, Pakistan. Isopropyl myristate (Sigma Aldrich, St. Louis, MO, USA), Carbopol 940 (Lubrizol, Wickliffe, OH, USA), methanol (Sigma Aldrich, St. Louis, MO, USA), acetonitrile (Sigma Aldrich, St. Louis, MO, USA), acetic acid (Sigma Aldrich, St. Louis, MO, USA), 2,2-diphenyl-1-picrylhydrazyl (DPPH) (Sigma Aldrich, St. Louis, MO, USA), gallic acid (Sigma Aldrich, St. Louis, MO, USA), ascorbic acid (Sigma Aldrich, St. Louis, MO, USA), *mushroom tyrosinase* (L.) (Sigma Aldrich. St. Louis, MO, USA), quercetin (Sigma Aldrich, St. Louis, MO, USA), Folin-Ciocalteu (Sigma Aldrich, St. Louis, MO, USA), ethanol (Merck, Darmstadt, Germany), Span 80 (Merck, Darmstadt, Germany), Tween 80 (Merck, Darmstadt, Germany), and triethanolamine (Merck, Darmstadt, Germany) were used in study. Distilled water was carried out in the Pharmaceutics laboratory of the Department of Pharmacy, IUB, Pakistan. All other organic solvents and chemicals used for extraction and analysis were of analytical and chromatographic grade.

### 2.2. Preparation of the Extract

Flaxseed was identified by the Taxonomist at the Cholistan Institute of Desert Studies of the Islamia University of Bahawalpur vide Ref. No.370/Botany, dated 3 April 2019. The preparation of the extract was performed as described by Khan & Akhtar [16] with some modifications. Seeds were ground in fine powder and were soaked into 80% ethanol for 72 h in a closed container, and the mixture was stirred at regular intervals (after every 6 h) for complete maceration followed by coarse filtration via 4 layers of muslin cloth. For fine filtration, this coarse filtrate was filtered using filter paper (Whatman No. 1) and subjected to a rotary evaporator under reduced pressure of 40 °C for solvent evaporation and concentration. The final extract was kept at 8 °C in a covered glass container for further studies; the same was repeated for the methanolic fraction. The final extract that was used in the formulation is free from the solvent.

### 2.3. Total Phenolic and Total Flavonoid Contents

Total phenolic content (TPC) of flaxseed extract was determined by the Folin–Ciocalteu method as reported previously; the results were expressed in mg of gallic acid equivalents (GAE)/g dry extract. Total flavonoid content (TFC) was determined using the aluminum chloride colorimetric method. The TFC results were reported as mg of quercetin equivalent (QE)/g dry extract [16].

### 2.4. Determination of Polyphenols by HPLC with UV Detection

The polyphenolic constitution of ethanolic seed extract was investigated according to Qayyum et al. [17]. Flaxseed extract (50 mg) was thoroughly mixed with 24 mL of methanol. Further, proceeded with the addition of 16 mL of distilled water. The final solution, after the addition of 10 mL of 6 M HCl, was kept at 95 °C for 2 h followed by filtration with a nylon membrane filter (0.45 µm). The analysis of flaxseed extract was performed on gradient HPLC (LC-10A, SHIMADZU, Kyoto, Japan). The chromatographic separation was carried out by employing a shim-pack CLC-ODS C-18 reverse phase column (250 × 4.6 mm, 5 μm). As eluent A: a mixture of water and acetic acid (≥99.5%) (94:6, *v*/*v*) at pH = 2.27 and eluent B: 100% acetonitrile were employed. Gradient elution program was 15% eluent B (0–15 min), 45% eluent B (15–30 min) and 100% eluent B (35–45 min) with the flow rate of 1 mL/min. Whole polyphenolic constituents in extract, including phenolic acids, were detected via an UV-visible detector (λ = 280 nm) and recognized by the comparison of UV-visible spectra of the peaks and the retention time obtained with the nine reference standards including quercetin, caffeic, gallic, vanillic, benzoic, *p*-coumaric, syringic, sinapic, and cinnamic acids. External calibration was performed for quantification [17].

### 2.5. Biological Activities

#### 2.5.1. Radical Scavenging Activity

Free radical scavenging activity of flaxseed extract was concluded using DPPH stable free radicals [18]. About 95 μL of 0.2 mM DPPH solution and 5 μL ethanolic extract (1 mg/mL) of flaxseed were mixed thoroughly in 96-well microplates and placed in the dark at 37 °C for 30 min. Ascorbic acid was used as standard, analytical grade methanol was used as control, and absorbance was recorded at 517 nm; the percentage of scavenging activity was measured by the following formula Equation (1):

DPPH scavenging activity (%) = [(*A*^0^ − *A*^1^)/*A*^0^] × 100 (1)

*A*^0^ = absorbance of the control and *A*^1^ = absorbance in the presence of a test or standard sample.

#### 2.5.2. Tyrosinase Inhibition Assay

Tyrosinase inhibition assay was carried out by adopting Ahmed’s method [19]. The positive control was kojic acid in the procedure, and enzyme inhibition was determined using Equation (2) below:

Inhibition (%) = (absorption of control − absorption of sample/absorption of control) × 100 (2)

#### 2.5.3. Determination of Sun Protection Factor Value of Extract

About 1 g of both methanolic and ethanolic extract were weighed separately, diluted with ethanol to the volume of 100 mL, and ultrasonicated for 5 min followed by filtration. The first 10 mL of the filtrate were rejected. Then, 5 mL aliquot was diluted with ethanol up to 50 mL. Then, 5 mL aliquot from this solution was diluted with ethanol to a final volume of 25 mL. The absorption data of this final solution was measured in the range of 290–320 nm after an interval of every 5 nm. Ethanol was used as blank. Results were recorded in triplicates by applying the Mansur equation, Equation (3) [20]:

(3) $$\mathrm{SPF}=\mathrm{CF}\;\times \;\sum_{320}^{290}\;\mathrm{EE}\;(\lambda )\;\times \;1\;(\lambda )\;\times \;\mathrm{Abs}\;(\lambda )$$

where, *CF* = correction factor (=10); *EE* (λ) = erythemal effect spectrum; 1 (λ) = solar intensity spectrum; *Abs* (λ) = absorbance of sunscreen product.

### 2.6. Formulation Development

#### 2.6.1. Construction of Phase Diagram

A pseudoternary phase diagram was built at ambient temperature by the water titration method [21]. Tween 80 and Span 80 were used in a 3:1 weight ratio as a mixed surfactant, and 40% ethanol in the aqueous phase was used as a cosurfactant along with isopropyl myristate (IPM) as oil. Mixed surfactant and isopropyl myristate (1 g) in different ratios (1:9, 3:7, 5:5, 7:3, and 9:1) was titrated against the aqueous phase (i.e., 40% ethanol) under moderate stirring using a magnetic stirrer. When equilibrium was achieved after stirring of 1 min the microemulsion was visually inspected for its transparency. A pseudoternary phase diagram was built using TernaryPlot.com (accessed on 25 January 2022).

#### 2.6.2. Preparation of Microemulsion

Upon identification of the microemulsion region, three compositions for placebo (P1, P2, and P3 (without extract)) and active (F1, F2, F3 (containing ethanolic flaxseed extract)) microemulsion were selected with increasing concentration of surfactant (Table 1). The microemulsion was formulated by dropwise adding hydroalcoholic aqueous phase in a mixture of oil, surfactant mix, and extract (in case of active microemulsions) at room temperature using a magnetic stirrer. The same procedure was repeated for placebo microemulsions but without adding extract.

#### 2.6.3. Determination of Zeta Size, Zeta Potential, and Polydispersity Index

Zeta size, zeta potential, and PDI of microemulsions were assessed using a nano zeta size, zeta potential, and PDI analyzer (ZS-90, Malvern Instruments, Malvern, UK) [16].

#### 2.6.4. Incorporation of Microemulsion in Gel

Carbopol 940 solution (1.5%) was prepared along with the addition of 0.1% methylparaben, and its pH was adjusted with triethanolamine [22]. Optimized microemulsions of placebo and active (a formulation containing extract) were thoroughly mixed with gel in 1:1 (*w*/*w*) separately to formulate placebo and active emulsiongels.

### 2.7. In-Vitro Evaluation of Microemulgels

#### 2.7.1. Organoleptic and Physical Stability Evaluation

Assessment of organoleptic (color and odor) and physical stability parameters including phase separation and liquefaction of all the samples of placebo and active microemulgels were carried out. Formulations were kept at specified temperature conditions, i.e., 8 °C, 25 °C, 40 °C, and 40 °C with 75% relative humidity (RH) for 3 months and evaluated for thermal stability at specified time intervals (0, 7, 14, 21, 28, 45, 60, and 90 days) [18].

#### 2.7.2. pH Measurement

The pH of freshly prepared placebo and active microemulgels were measured using a digital pH meter (WTW, Inolab pH7110, Benchtop Meter, Weilheim, Germany). The samples were kept in specified temperature conditions, i.e., 8 °C, 25 °C, 40 °C, and 40 °C with 75% RH for 3 months for further pH studies at specified time intervals; readings were recorded in triplicates for the entire study span.

#### 2.7.3. Electrical Conductivity Determination

A digital conductivity meter (WTW COND-197i, Weilheim, Germany) was employed in the study to record the electrical conductivity. All samples were kept in specified temperature conditions, i.e., 8 °C, 25 °C, 40 °C, and 40 °C with 75% RH for electrical conductivity at a specified time interval and the results were recorded in triplicates for the entire study span of three months.

#### 2.7.4. Rheology

The rheology of all samples of the finalized formulation kept at specified temperature conditions (for 3 months) was studied at 25 ± 0.5 °C with the help of a programmable Brookfield Rheometer (Model DV-III ultra, Brookfield, IL, USA). Parameters studied included shear rate, shear stress, and viscosity, and the results were obtained in triplicate with the help of Rheocalc Version 2.6.

### 2.8. In Vitro Release Comparison of Microemulgel and Microemulsion

Drug release studies were performed to compare the release from microemulsion and microemulgel having the same (4%) extract concentration by adopting Froelich’s method with some modifications [11]. For this purpose, a 15 mm Franz diffusion cell (PERME GEAR Inc#4G-01-00-15-12) each containing 12 mL receptor volume capacity filled with phosphate buffer solution (pH = 5.5) was used. The Franz cells were equipped with a cellophane dialysis tubing membrane (MWCO 12—14,000 Da) having a surface area of 1.76 cm^2^, which was soaked for 24 h in an acceptor medium at 32.0 ± 0.5 °C, prior to use in studies. Samples (0.5 g) of each microemulsion and microemulgel were placed and spread evenly on the membrane via the donor compartment. During the study period of 12 h, the receptor fluid was kept stirring at 200 rpm and its temperature was sustained at 32.00 ± 0.50 °C. Samples (1 mL) were periodically drawn at fixed interims (i.e., 0, 0.25, 0.5, 1, 2, 3, 4, 6, 8, 10, and 12 h) for 12 h from the receptor compartment and to maintain the sink conditions, instantly replaced with a fresh and equal amount of receptor. Withdrawn samples were analyzed using a UV-visible spectrophotometer and the amount of cumulative drug release versus time graph of microemulsion and microemulgel was plotted. Kinetic modeling of the release data was executed by the application of different kinetic models, i.e., zero-order, first-order, Higuchi, and Korsmeyer Peppas on the release data.

### 2.9. In Vivo Evaluation of Microemulgels

#### 2.9.1. Study Design and Ethical Approval

In vivo evaluation of microemulgel was carried out for the period of 12 weeks and included 13 healthy Asian female volunteers with ages ranging from 25–40 years. It was a single-blind, monocentric and placebo-controlled study, performed after approval from the Board of Advanced Study and Research (BASR) and the institutional ethics committee, Faculty of Pharmacy, The Islamia University, Bahawalpur with reference No. 116-2021-/PHEC and proceeded according to the Declaration of Helsinki. Written consent from all female volunteers was obtained after a thorough examination of their skin for any severe skin disease and informing them about all protocols, objectives, and possible adverse effects. Volunteers having pregnancy, hypersensitivity, excessive facial hairs, any skin disease, the habit of smoking, noncompliance behavior, having any skin treatment or steroidal therapy, and participating in another similar study were not included in the study [18,23,24].

#### 2.9.2. Panel Test

Sensory evaluation of formulations was carried out in terms of ease of application, irritation, shine, sense in long term (means feeling after a long time of application, 90 days), feel after application, sense of softness (means a feeling of softness on the skin after application of microemulgel), and spreadability by panel test, prior to in vivo study. The panel of thirteen participants was provided with placebo and active formulations and ask them to score from 0–5 after rubbing between fingers [24].

#### 2.9.3. Patch Test

A patch test was conducted prior to the start in vivo study and the skin of volunteers (*n* = 13) was evaluated for any hypersensitivity by measuring melanin and erythema levels before and after 48 h of application of the patch as previously described by Huma et al. in their study [18].

#### 2.9.4. In Vivo Evaluation

In vivo studies were carried out for 12 weeks and parameters evaluated in this study were skin melanin, erythema, sebum, moisture, and elasticity. All these parameters were measured by using a mexameter, sebumeter, corneometer (i.e., by Cutometer^®^ MPA 580 C&K, Electronic GmbH, Koln, Germany), and elastometer by MPA 580 C&K Electronic GmbH, Koln, Germany.

### 2.10. Statistical Analysis

Calculations were performed in triplicates and results were expressed as mean ± S.D. Data obtained during the whole study period was statistically evaluated using SPSS version 23.0. Paired sample *t*-test was applied to compare the results of placebo and active microemulgels. *p*-Values less than 0.05 defined the results as statistically significant. Double Dummy Solver software (DDSolver, version 12.99X) was used for the analysis of drug release data. GraphPad Prism version 8.4.3 was used to draw the graphs.

## 3. Results

### 3.1. Phytochemical Screening

The TPC value of methanolic and ethanolic extracts of flaxseed was 22.30 ± 0.35 mg GAE/g of dry weight and 18.75 ± 0.44 mg GAE/g of dry weight, respectively, as shown in Table 2. The TFC content was 1.28 ± 0.018 mg QE/g of dry weight and 1.34 ± 0.021 mg QE/g of dry weight of methanolic and ethanolic extracts, respectively (Table 2).

**Figure 1:**
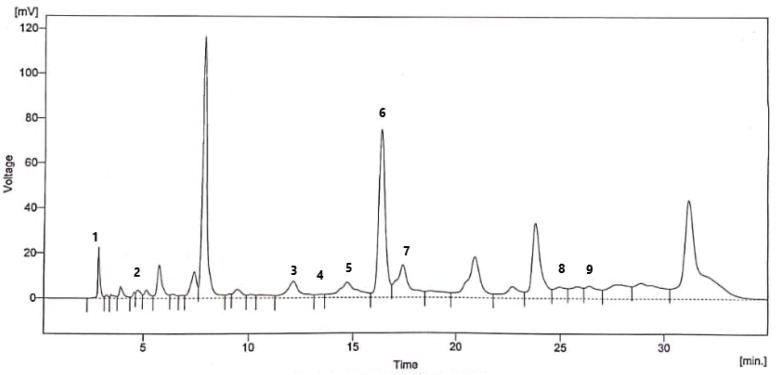
HPLC-UV chromatogram of the ethanolic flaxseed extract at λ = 280 nm. Peak numbers refer to Table 3.

In the present study, the ethanolic extract of flaxseed was evaluated using HPLC-UV for the existence of polyphenols against nine reference standards, i.e., quercetin, caffeic, gallic, vanillic, benzoic, *p*-coumaric, syringic, sinapic, and cinnamic acids using HPLC-UV. Since the assays for biological activities and TFC of ethanolic extract gave greater results as compared to methanolic extract, ethanolic extract is selected for HPLC-UV and formulation development. Retention times of unknown polyphenols present in flaxseed extract were compared with standard polyphenolic compounds for validation and confirmation. The amount of the polyphenolics is estimated by comparing the peak area (Figure 1) with a constructed calibration curve. Relative retention times in a sample are shown in Table 3. A major phenolic component in ethanolic flaxseed extract was benzoic acid (44.22 µg/g), followed by syringic acid (41.82 µg/g), caffeic acid (14.44 µg/g), quercetin (8.21 µg/g), *p*-coumaric acid (7.88 µg/g), cinnamic acid (7.35 µg/g), sinapic acid (3.35 µg/g), vanillic acid (2.58 µg/g), and gallic acid (2.17 µg/g).

### 3.2. Biological Activities

#### 3.2.1. Radical Scavenging Activity

In the current research study, the antioxidant activity of flaxseed extracts is ascertained via the 2,2-diphenyl-1-picrylhydrazyl (DPPH) method [25,26]. Current research work revealed that the DPPH radical scavenging activity of flaxseed methanolic and ethanolic extracts were 86.44 ± 0.73% and 87.00 ± 0.52%, respectively, as shown in Table 4.

#### 3.2.2. Tyrosinase Inhibition Assay

The current study involves the investigation of methanolic and ethanolic extracts of flaxseed for inhibitory activity against tyrosinase and it showed 68.77 ± 0.67% and 72.00 ± 0.55% inhibition, respectively (Table 4).

#### 3.2.3. Determination of Sun Protection Factor Value of Extract

The SPF of methanolic and ethanolic extracts of flaxseed was 19.30 ± 0.06 and 21.80 ± 0.10, respectively (Table 4).

### 3.3. Formulation Development

#### 3.3.1. Construction of Phase Diagram

**Figure 2:**
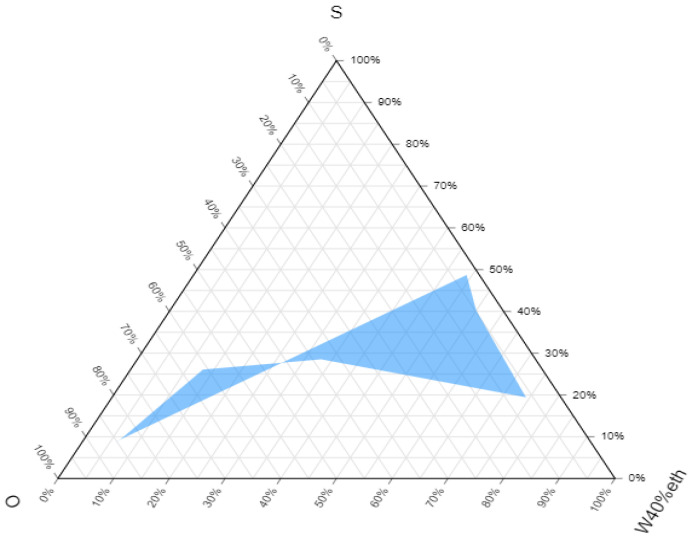
Ternary phase diagram of microemulsion composed of oil (O), surfactant (S), and aqueous phase (W, ethanol (40%)).

Initially, a pseudoternary phase diagram was constructed successfully with ethanol as a cosurfactant, as reported earlier that short-chain alcohols promote the formulation of microemulsion [21]. The use of mixed surfactants gave a hydrophilic-lipophilic balance (HLB) value of 12.325, which is within the acceptable range of HLB value (9–17) for surfactants to formulate microemulsions [27]. Figure 2 indicates the pseudoternary phase diagram of microemulsion composed of oil (O), surfactant (S), and aqueous phase (W). The shaded region of Figure 2 indicates the transparent microemulsion region while the rest of the region indicates conventional or turbid emulsions.

#### 3.3.2. Preparation of Microemulsion

Three different compositions from the pseudoternary phase diagram were selected each for placebo (P1, P2, P3) and active (F1, F2, F3) microemulsions and were further assessed using zeta size, zeta potential and polydispersity index (PDI).

#### 3.3.3. Determination of Zeta Size, Zeta Potential and Polydispersity Index

**Figure 3:**
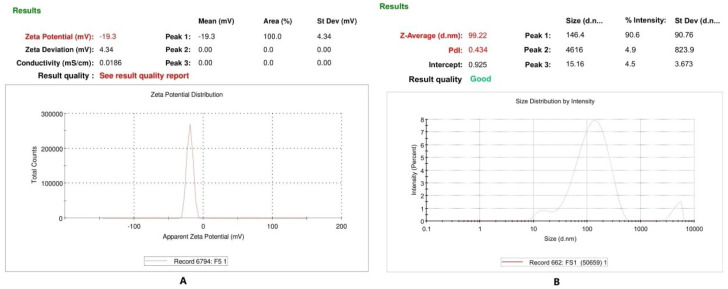
Zeta potential (**A**), zeta size, and PDI (**B**) of optimized microemulsion F3.

Table 5 depicts that the size of microemulsion globules increases with a decrease in surfactant concentration. P3 and F3 with the lowest globule size of 179.00 and 99.22 nm, respectively, were selected for incorporation in gel (microemulgel formulation). Moreover, the PDI for both P3 and F3 formulations was less than 0.50. The zeta potential of all formulations ranged from −16.10 to −19.30 mV. In addition, Figure 3 shows the zeta potential, zeta size, and PDI of optimized microemulsion F3.

### 3.4. Incorporation of Microemulsion in Gel

pH adjustment of plain gel was carried out within the average acceptable skin pH range (i.e., 5–6) using triethanolamine, which also increases the consistency of carbopol solution and gives it a gel-like appearance [18,21]. Microemulsions P3 and F3 were successfully incorporated into gel to obtain placebo (P; without extract) and active (F; with 4% extract) microemulgels, respectively.

### 3.5. In Vitro Evaluation of Microemulgels

#### 3.5.1. Organoleptic Evaluation

The results of the organoleptic evaluation are shown in Table 6. There was no change in odor, phase separation, or liquefaction in any of the placebo and active formulations stored at predefined temperature conditions except a slight change in color of active formulations stored at 40 °C and 40 °C with 75% RH at the end of the study period.

#### 3.5.2. pH Measurement

**Figure 4:**
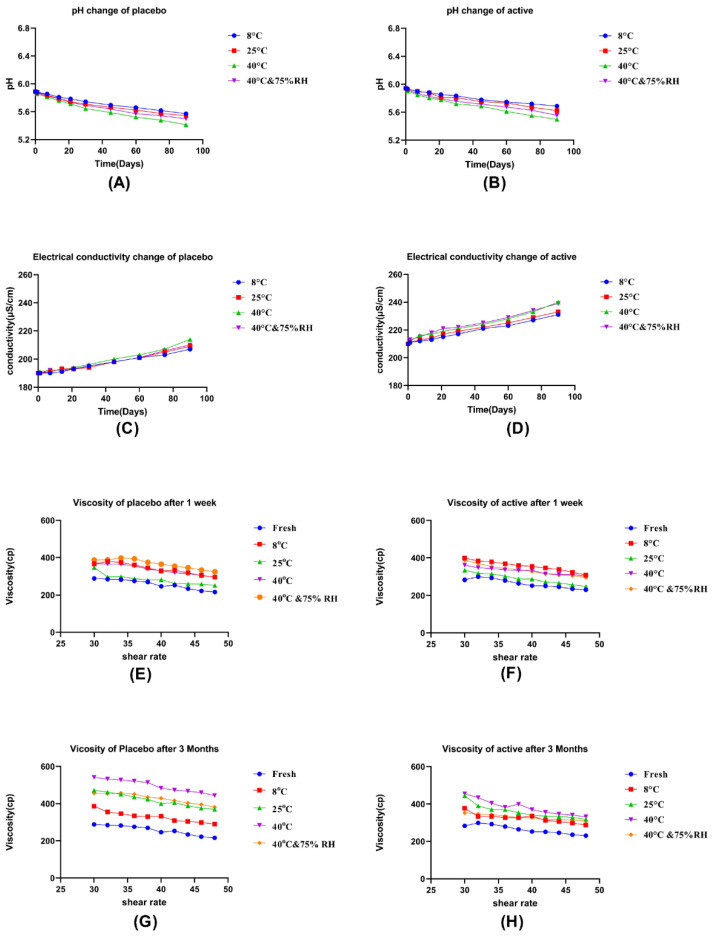
In vitro evaluation for placebo and active microemulgels kept at specified temperature conditions. (**A**,**B**), pH change; (**C**,**D**), electrical conductivity change; (**E**,**F**), viscosity change (a week); (**G**,**H**), viscosity change (3 months) for placebo and active microemulgels, respectively.

The pH of both placebo and active formulations was 5.887 and 5.942, respectively, when prepared freshly. A small decline in pH was observed for both placebo and active formulations under predefined temperature conditions for 3 months as shown in Figure 4A,B. Results of paired sample *t*-test showed a significant difference between the pH of placebo and active formulation.

#### 3.5.3. Electrical Conductivity Determination

The conductivity of newly formulated placebo and active formulations was 190 μS/cm and 210 μS/cm, respectively. Over the whole course of the study, under all conditions of storage, a minor surge in conductivity values was noticed for both formulations (placebo/active) as shown in Figure 4C,D. A slightly higher increase in conductivity values was noticed for formulations observed under 40 °C and 40 °C + 75% RH than that of others. On application of paired *t*-test, a significant difference was observed between the conductivity of placebo and active formulations.

#### 3.5.4. Rheology

In the present study, the change in viscosity was observed during the application of shear stress. Viscosity at steady shear started to decrease gradually with a progressive increase in applied shear stress in all samples of placebo and active formulations during the whole study period (Figure 4E–H).

### 3.6. In-Vitro Release Comparison of Microemulsion and Microemulgel

**Figure 5:**
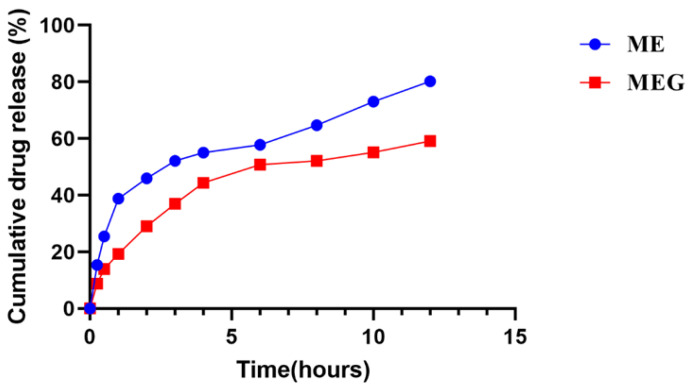
Comparison of (%) cumulative drug release from microemulsion and microemulgel after 12 h. ME is a code representing microemulsion and MEG is a code used for microemulgel in release studies.

The results of the in vitro release of flaxseed bioactive components from microemulsion (ME) and microemulgel (MEG) were shown in Figure 5. The percent cumulative drug release of ME and MEG was 80% and 59%, respectively, during the 12 h of the study. Both ME and MEG showed nonlinear biphasic drug release. Drug release for ME was initially rabid, about 56% during the 4 h of study, and then slowly up to 80% in the next 8 h. MEG released about 43% drug in the first 4 h of the study, and about 59% of the drug was released slowly in the next 8 h of the study. There was a prominent difference in release rate between the ME and MEG, as ME shows greater release in 12 h.

#### Kinetic Modeling of Release Data

The data obtained from in vitro release studies was further analyzed by putting them in different kinetic models as shown in Table 7. Closer to 1 coefficient of determination (R^2^) values for ME and MEG were 0.9789 and 0.9813, respectively, at pH 5.5. The diffusion exponent (*n*) was 0.420 and 0.334 for ME and MEG, respectively.

### 3.7. In Vivo Evaluation of Microemulgels

#### 3.7.1. Panel Test

**Figure 6:**
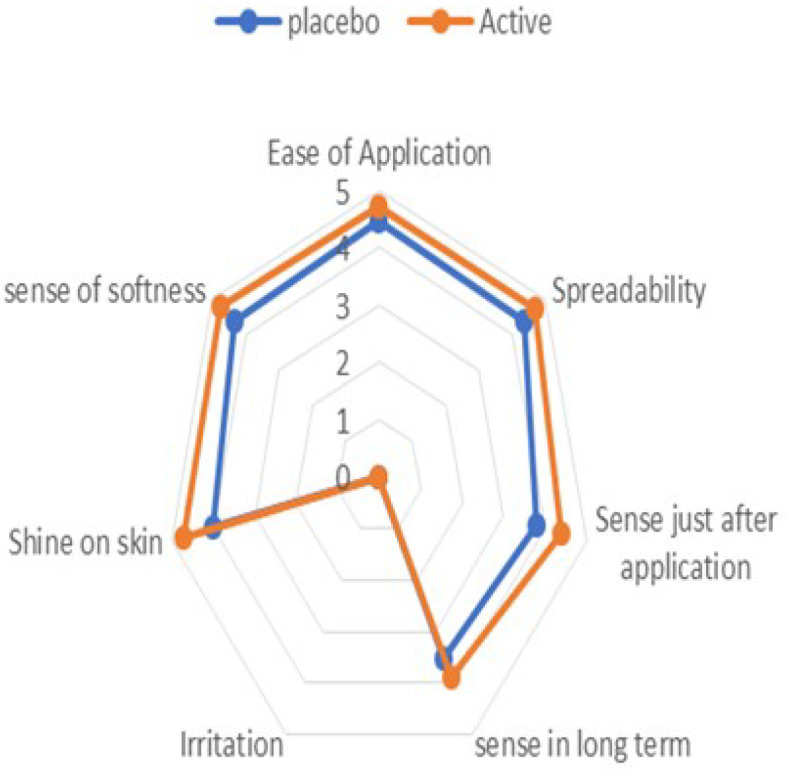
Panel test of placebo and active on human volunteers (*n* = 13).

In the panel test, volunteers were asked to fill the questionnaires, presented with seven important sensory parameters of microgels. The results obtained were presented in terms of average points on the opinion of each volunteer as indicated in Figure 6; there was good spreadability, softness, and no irritation on an application for placebo and active microemulgels.

#### 3.7.2. Patch Test

**Figure 7:**
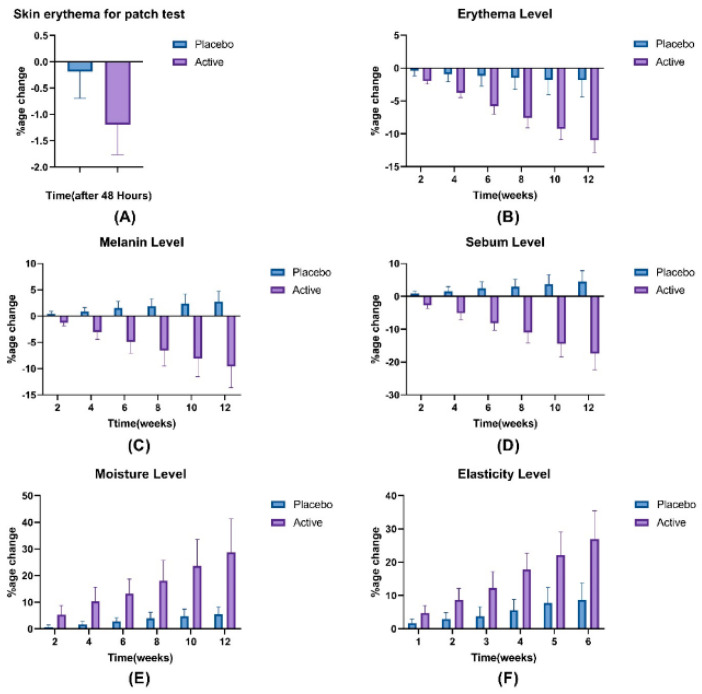
In vivo evaluation of placebo and active microemulgels. (**A**), skin erythema; (**B**), erythema level; (**C**), melanin level; (**D**), sebum level; (**E**), moisture level; (**F**), elasticity level.

Prior to the noninvasive in vivo study, cosmetic products are importantly analyzed for any skin irritation or other harmful effect by applying of patch test on selected human volunteers for 48 h [28]. Results of the patch test in the current study did not show an increase in erythema level of any volunteer (Figure 7A), leading to the confirmation of microemulgel (both placebo and active) safety and suitability for its topical use in selected volunteers.

#### 3.7.3. Skin Erythema Level

Erythema level at the site of placebo and active microemulgels application of volunteers was measured regularly on 0, 2, 4, 6, 8, 10, and 12 weeks. Results indicated a slight and pronounced decrease in skin erythema level throughout the study period for placebo and active formulations, respectively, as shown in Figure 7B. The gradual decrease in erythema for active was tenfold greater than that of the placebo. On application of the ANOVA test, it was found that both placebo and active formulations produced significant effects over time. On application of paired sample *t*-test, a significant difference was observed between the erythema effects produced by placebo and active formulations.

#### 3.7.4. Skin Melanin Level

Melanin level measured for placebo microemulgel slightly increased during the study period. While the constant and prominent decrease in melanin level for active microemulgel was observed throughout the study period (Figure 7C). On application of ANOVA, significant effects were observed during the whole study span in melanin levels for placebo and active formulations. After application of paired sample *t*-test, a significant difference was observed between the melanin level induced by placebo and active.

#### 3.7.5. Skin Sebum Level

Sebum level for placebo and active microemulgels was monitored for 12 weeks after an interval of every 2 weeks. Results indicated a slight and irregular increase (4.5%) in sebum level for placebo formulation. While for active microemulgel, a prominent decrease of up to 17.0% was observed for the whole study period of 12 weeks (Figure 7D). When statically analyzed via ANOVA, it was observed that both placebo and active showed significant effects over time. A significant difference was observed between the effects of both; placebo and active formulations on the application of paired sample *t*-test.

#### 3.7.6. Skin Moisture Level

Skin hydration level was monitored for 12 weeks and obtained results indicated an increase in moisture level for both placebo and active microemulgels. However, this increase was irregular and slight for placebo, i.e., 5.5% as compared to active, which showed a constant and 28% increase in moisture level (Figure 7E). By analyzing with ANOVA, it was observed that placebo and active formulations produced significant moisturizing effects over time. When applied paired sample *t*-test, there was a significant difference in the moisturizing effects of placebo and active formulations.

#### 3.7.7. Skin Elasticity

Results of skin elasticity for placebo and active indicated the increase in elasticity for both placebo and active. However, an increase in elasticity for the active formulation was regular and more pronounce, i.e., 27.0% as compared to an increase in elasticity for placebo, i.e., 8% (Figure 7F). According to ANOVA, there was a significant change in elasticity produced by placebo and active formulations for the whole study span. According to paired sample *t*-test, there was a significant difference in elasticity produced by placebo and active formulations.

## 4. Discussion

Phenolic acids are naturally hydrophilic antioxidants, comprised of benzoic acid and cinnamic acid derivatives and are widely present in vegetables, aromatic herbs, fruits, and spices [29]. Prior studies have confirmed that flaxseeds have considerable phenolic contents, i.e., 20.20 mg GAE/g in 80% methanolic extract, and 32.60 mg GAE/g in 80% ethanolic extract [6]. In another study, TPC in flaxseed was 16.70 mg GAE/g, which is almost near to the results of the present study [6,30]. Flavonoids are widely present in the kingdom Plantae and exert antioxidant activity along with other biological activities [29,31]. Previous studies also reported a significant amount of flavonoids in flaxseed [31]. Small variations in results may be associated with many factors including the variety of flaxseed used for extraction, extraction technique, temperature, and solvent used for extraction [6]. Early studies confirm the presence of phenolic components including quercetin, caffeic, gallic, vanillic, benzoic, *p*-coumaric, syringic, sinapic, and cinnamic acids in flaxseed [32]. Qayyum et al. reported earlier that the amount of phenolic components may vary in plants, and there is a significant positive correlation between phenolic compounds and antioxidant potential [17]. So, ethanolic flaxseed extract may be a promising candidate in skincare products as potential segment showing a large number of phenolic contents with significant antioxidant activity and tyrosinase inhibition as well as good SPF.

The presence of phenols in plants is the main cause of the antioxidant potential of plants, which are potent free radical scavengers and reducing agents [6,33]. The antioxidant activity of flaxseed extracts in present studies is in line with prior studies [6] and is characterized by the existence of flavonoids and greater contents of phenolic acids and lignans in flaxseed [32].

The major enzyme for melanogenesis in epidermal layers is tyrosinase (phenol oxidase), and a variety of tyrosinase inhibitors are developed and introduced in cosmeceutical preparations to minimize the excessive production of melanin [34]. The mechanism of *mushroom tyrosinase* assay involves the formation of L-dopa as a result of L-tyrosine hydroxylation [35]. Lignans, phenolic acids, and flavonoids act as tyrosinase inhibitors, and some flavonoid derivatives from herbal or synthetic sources have potent tyrosinase inhibition activity [36]. The promising tyrosinase inhibition activity of flaxseed extract may be due to phenols, lignans, and flavonoids, which have established antityrosinase potential [32].

SPF is a proportion of least erythema dose on unprotected skin to least erythema dose on protected skin. A higher SPF value indicates more protection from the sun [20]. Antioxidants provide great protection to skin from damage caused by ultraviolet rays from the sun. It is reported earlier that vitamin E, vitamin C, tannins, phenols, and flavonoids are good candidates to provide sun protection [18,37]. Flaxseed extracts can protect the skin against ultraviolet rays 19.3 and 21.8 times more than that of unprotected skin and can be a good candidate for skincare products. Moreover, this ability of flaxseed may be attributed to the presence of phenolic compounds, lignans, and vitamins C and E [5].

The size of microemulsion globules in the present work increases with a decrease in surfactant concentration as reported [38]. The globule size of the selected microemulsion for further proceeding was within the range given in the literature [10]. PDI indicates whether the size of particles or globules in a formulation is uniform or not as a PDI value less than 1 indicates uniformity in size [24]. PDI for placebo and active microemulsions selected for microemulgel formulation was less than 0.5 which ensures the narrow size distribution of oil globules as illustrated by Kumar & Sinha [39]. Zeta potential ensures the electrostatic stability of nanosize droplets of the formulations [24]. The negative zeta potential in the present research is due to the existence of ethanol as it induces the negative charge on globules of the system [40]. The high zeta potential of the system is due to the high concentration of ethanol, which increases the stability of the system by increasing the electrostatic repulsion and avoiding the formation of agglomerates [41].

In the present work, findings of the organoleptic evaluation of placebo and active formulations ensured that they were stable while slight variations in the color may be due to the oxidation reaction at accelerated conditions [42]. According to the literature, skin pH varies from 4.0–7.0, but for cosmeceutical evaluation, pH 5.0–6.0 is considered as the best and the average pH of skin [18]. In the current study, although there was a decrease in the pH for both placebo and active formulations, it remained inside the average acceptable range of skin pH. The reason for the decline in the pH of both formulations might be the degradation of Tween 80, as its degradation involves hydrolysis and oxidation eventually leading to the formation of fatty acid, aldehydes, peroxides, epoxides, and ketones, which in turn decrease the pH of a system [43]. Another reason for the decline in the pH of active microemulgel under accelerated conditions of storage is associated with the oxidation of oils present in the extract which leads to the formation of aldehydes and peroxides [44]. As reported earlier in a similar study of extract-based formulation, the acidic nature of extracts may also be the reason for the decline in the pH of active microemulgel [16].

Conductivity is an imperative parameter for the assessment of physical changes like stability at different conditions of storage [45]. The reason for the increase in conductivity is storage at accelerated temperature as it is reported earlier that an increase in temperature induces a positive change in conductivity [46]. It is evident from the literature that an increase in conductivity is a clustering of droplets of microemulsion along with an exchange of charges among aggregate [47]. Furthermore, the coalescence of oil droplets might be the reason for increased conductivity [48]. There was no considerable increase in conductivity values in the present study, so it can be considered that placebo and active microemulgels were stable in the studied conditions [45].

The most important parameter of topical semisolid formulations to assess their physical stability and behavior during use is rheology [23]. In the present work, the decrease in viscosity with an application of shear stress on placebo and active formulation shows that they have non-Newtonian shear thinning behavior. A gel is a cross-linked network of entangled polymer chains, which is the reason to increase the viscosity of product containing gel, but with the application of shear stress, these polymeric chains align themselves in the direction of flow leading to a decrease in viscosity of the system. This shear-thinning behavior is important for cosmetic products, as it is closely related to spreadability and enables the product to spread smoothly and form an even and thin layer on the skin when applied in one direction [49].

In vitro release pattern is an important parameter, as it is helpful to determine drug diffusion across the membrane, which is affected by various factors including; interaction between components of formulation and drug, physicochemical properties of ingredients, and internal structure of a vehicle [50]. The slow release of the drug (flaxseed bioactive components) from MEG may be attributed to the high viscosity of MEG as compared to ME, owing to the carbopol gel. Earlier research studies have confirmed that in vitro drug release decreases with the increase in the viscosity of formulations [51,52]. Another reason for slower release from MEG may be the restructuring of gel, which results in the extension of the diffusion pathway for active moiety by reducing the diffusion area [52]. On application of paired sample *t*-test, results show a significant difference in drug release from microemulsion and microemulgel.

In accordance with the results of kinetic modelling, both flaxseed extract-loaded microemulsion and microemulgel followed the Korsmeyer Peppas model (Kp), as in the case of the former model, the coefficient of determination (R^2^) values was closer to 1 as compared to R^2^ values obtained after application of other models. Following of Kp model by ME and MEG, we declare them a diffusion-controlled delivery system [50,52]. In the case of MEG, carbopol gel is the reason for controlled release, as it is reported earlier that gels are controlled released formulations [15]. Moreover, the diffusion exponent (*n*) values indicate that drug release from both formulations followed the Fickian diffusion mechanism [39].

In the panel test, both the active and placebo microemulgels provided softness to the skin which may be associated with isopropyl myristate [53]. The active formulation has a little more softness due to the presence of polyphenols existing in flaxseed extract [54].

The safety of any cosmetic product is analyzed by evaluating its tendency to cause dermatitis upon application on the skin [55]. The application of cosmetic products must not cause any type of inflammation especially contact dermatitis, which results due to the skin toxicity of chemicals used in the preparation of cosmetic products [54]. Flaxseed contains a significant amount of phenolic compounds, polyunsaturated fatty acids (omega-3), and secoisolaiciresinol diglucoside (SDG) [5]. A noticeable decrease in erythema on the application of active microemulgel may be due to phenolic compounds and SDG present in flaxseed, which is responsible for anti-inflammatory properties [56,57] Another possibility for a decrease in erythema is the presence of omega-3 fatty acids in flaxseed, as it is reported earlier that polyunsaturated fatty acids decrease skin inflammation especially caused by ultraviolet rays [58].

Whitening of the skin can be achieved by inhibiting of tyrosinase pathway or reducing ROS [59]. Tyrosinase is an enzyme in humans which helps in synthesizing a pigment called melanin, responsible to impart skin color. Overactivity of tyrosinase causes hyperpigmentation while underactivity of this enzyme leads to the depigmentation of melanin in the skin. Decreased level of ROS in melanocytes also prevents the production of melanin in the skin. Flavonoids and phenolic compounds have ability to reduce the melanin level in the skin. The potential of polyphenols to inhibit tyrosinase enzyme and scavenge ROS reduces the production of melanin in skin eventually leading to fair skin [55,59].

In the current research work, a decrease in melanin level after application of active microemulgel may be associated with the presence of phenolic components in flaxseed which has antityrosinase as well as antioxidant activity [32,59]. Previous studies also confirm that flavonoids in extract-based formulations decrease skin melanin levels [55].

Sebaceous glands are associated with hair follicles and present all over the body except palms and soles. They produce a lipid-rich secretion, “sebum” [55,60]. Sebum plays an important role of skin protection by lubricating it during cold environments [54]. Excessive secretion of sebum causes pore enlargement, which leads to many pathological conditions of the skin, including acne vulgaris due to microbial infection [18]. Reduction in sebum level after application of active microemulgel is associated with antioxidants, flavonoids, and α-linolenic acid present in flaxseed. It is reported earlier that antioxidants including flavonoids reduce sebum production by scavenging free radicals [54,55]. The presence of α-linolenic acid in flaxseed may also be the reason of sebum reduction as reported earlier [60]. In the current study, an increase in skin moisture level by the application of active microemulgel may be associated with the presence of flavonoids and tocopherols in flaxseed. Previous studies have confirmed that flavonoids provide photoprotection to skin and help to recover skin in terms of functions and structure by improving its moisture level [55]. Tocopherol also improves the skin by enhancing the hydration capacity of the skin, eventually leading to increased moisture levels [61].

Elastin and collagen are the main components of skin, responsible to provide skin with elasticity and integrity. Degradation of these components results in skin aging [62,63]. Skin elasticity can be negatively affected by the production of elastase and collagenase as a result of skin exposure to ultraviolet rays [55,63]. Skin can be protected from photoaging by using of antioxidants [55]. An increase in skin elasticity by active microemulgel may be attributed to the antioxidants, including polyphenols, vitamin C, and tocopherol present in flaxseed [5]. As it was reported earlier, polyphenols, vitamin C, and tocopherol help to decrease skin roughness and photo-damaging by protecting them from free radicals [61].

## 5. Conclusions

Studied extracts have shown prominent results for both ethanolic and methanolic extracts of flaxseed, and HPLC-UV analysis showed a significant extent of phenolic contents ultimately proving it a suitable candidate for the cosmetic industry. A stable microemulgel was successfully formulated by selecting the most suitable available components and ethanolic extract of flaxseed. Bioactive components release studies proved the flaxseed extract containing microemulgel as a controlled release formulation. After confirming in vitro stability it was further evaluated for in vivo studies. Results of in vivo studies showed that active formulation recovered the skin from any ultraviolet rays-related imperfections in terms of erythema, melanin, sebum level, moisture, and elasticity. On application of ANOVA on change in all skin parameters, a significant (*p* < 0.05) difference was observed proving it an effective cosmeceutical product. In future research, it should be clinically tested on different skin diseases such as eczema and psoriasis. Flaxseed extract can also be evaluated for more biological activities, toxicity, and bioavailability and its uses in other pharmaceutical products.

## Figures and Tables

**Table 1:** Composition of placebo and active microemulsions.

Microemulsion (Codes)	Surfactant Mix (Polysorbate 80 + Sorbitan Oleate, 3:1) * (%, *w*/*w*)	Oil (Isopropyl Myristate) * (%, *w*/*w*)	Aqueous Phase (%, *w*/*w*)	Ethanolic Extract (%, *w*/*w*)
P1	25	15	60	---- ^a^
F1	25	15	56	4
P2	30	15	55	---- ^a^
F2	30	15	51	4
P3	35	15	50	---- ^a^
F3	35	15	46	4

* According to International Nomenclature of Cosmetic Ingredients. ^a^ As it is a placebo, there is no extract used.

**Table 2:** Total phenolic content (TPC) and total flavonoid content (TFC) of the extracts prepared from flaxseed (mean ± S.D., *n* = 3).

Extract of Flaxseed	TPC (mg GAE/g Extract)	TFC (mg QE/g Extract)
Methanolic extract	22.30 ± 0.35 ^a^	1.28 ± 0.018 ^a^
Ethanolic extract	18.75 ± 0.44 ^b^	1.34 ± 0.021 ^a^

GAE, gallic acid equivalents; QE, quercetin equivalent. Values with the different superscript letters (within a column) are significantly different.

**Table 3:** Polyphenolic constituents (µg/g) of the ethanolic flaxseed extract determined by HPLC-UV at λ = 280 nm.

Peak No.	Retention Time (min)	Compounds Identified	Amount in Dried Extract (μg/g)
1	2.807	Quercetin	8.21
2	4.713	Gallic acid	2.17
3	12.127	Caffeic acid	14.44
4	13.473	Vanillic acid	2.58
5	14.713	Benzoic acid	44.22
6	16.380	Syringic acid	41.82
7	17.420	*p*-Coumaric acid	7.88
8	24.973	Cinnamic acid	7.35
9	26.400	Sinapic acid	3.35

**Table 4:** DPPH radical scavenging activity (%), tyrosinase inhibition (%), and sun protection factor (SPF) of the extracts prepared from flaxseed (mean ± S.D., *n* = 3).

Extract of Flaxseed	DPPH Radical Scavenging Activity (%)	Tyrosinase Inhibition (%)	SPF
Methanolic extract	86.44 ± 0.73 ^b^	68.77 ± 0.67 ^b^	19.30 ± 0.06 ^b^
Ethanolic extract	87.00 ± 0.52 ^a^	72.00 ± 0.55 ^a^	21.80 ± 0.10 ^a^

DPPH, 2,2-diphenyl-1-picrylhydrazyl; SPF, sun protection factor. Values with the different superscript letters (within a column) are significantly different.

**Table 5:** Characterization of placebo and active microemulsions.

Microemulsion (Codes)	Globule Size (nm)	Zeta Potential (mV)	Polydispersity Index
P1	199.00	−16.70	0.551
F1	239.20	−16.10	0.307
P2	192.90	−16.80	0.578
F2	236.80	−19.10	0.383
P3	179.00	−16.70	0.431
F3	99.22	−19.30	0.434

**Table 6:** Organoleptic evaluation of fresh formulations and after 90 days at different temperatures.

Observed Parameter	Fresh		After 90 Days							
			8 °C		25 °C		40 °C		40 °C ± 75% RH	
	P	F	P	F	P	F	P	F	P	F
Color	wt	owt	wt	owt	wt	owt	wt	Dowt	wt	Dowt
Odor	-	-	-	-	-	-	-	-	-	-
Phase separation	NA	NA	-	-	-	-	-	-	-	-
Liquefaction	-	-	-	-	-	-	-	-	-	-

RH, relative humidity; (P) placebo microemulgel; (F) active microemulgel; (NA) not applicable; (-) absent; (wt) whitish; (owt) off white; (Dowt) dark off white.

**Table 7:** Kinetic modeling of release data of microemulsion (ME) and microemulgel (MEG).

Formulation	Zero Order (R^2^)	First Order (R^2^)	Higuchi (R^2^)	Kp (R^2^)	*n*
ME	0.5768	0.813	0.965	0.9789	0.420
MEG	0.3095	0.7296	0.9019	0.9813	0.334

R^2^, coefficient of determination; Kp, Korsmeyer Peppas; n, release exponent of Kp model; ME, flaxseed extract containing microemulsion; MEG, flaxseed extract containing microemulgel.

## Data Availability

Not applicable.
